# Progress of long-chain noncoding small nucleolar RNA host genes in thyroid cancer

**DOI:** 10.3389/fonc.2025.1570684

**Published:** 2025-10-29

**Authors:** Tingyu Yang, Yuanxu Xie

**Affiliations:** Bishan Hospital of Chongqing Medical University, Bishan Hospital of Chongqing, Chongqing, China

**Keywords:** long noncoding RNA, nucleolar RNA host genes, thyroid carcinoma, competitive endogenous RNA, epithelial-mesenchymal transition

## Abstract

Small nucleolar RNA host genes (SNHGs) are a class of long non-coding RNAs that are widely aberrantly expressed in thyroid cancer and regulate tumor progression through the “SNHG–miRNA–signaling pathway” network. SNHG7 promotes cell proliferation and metastasis via the PI3K/Akt pathway and correlates with radioactive iodine resistance; SNHG15 accelerates epithelial-mesenchymal transition via the Hippo-YAP1 pathway; SNHG12 drives malignant phenotypes by interacting with Wnt/β-catenin signaling; SNHG14 enhances tumor invasion through multiple miRNA axes. In contrast, SNHG3 and SNHG5 exhibit tumor-suppressive effects in some studies. Overall, SNHGs may serve as molecular biomarkers and hold potential therapeutic target value. However, existing evidence is largely based on *in vitro* and small-sample studies, requiring further validation in clinical cohorts and functional models.

## 1 Introduction

Thyroid cancer is the most common endocrine-related malignancy. Most patients achieve favorable outcomes through conventional treatments such as surgery (1). However, certain thyroid cancers exhibit more aggressive features—including neurovascular invasion, local recurrence, and distant metastasis—which respond poorly to standard therapies and ultimately compromise patient survival (2–4).

Within the broader context of oncology, discoveries in targeted and immunotherapy have brought landmark breakthroughs to cancer treatment, with RNA-targeting strategies emerging as a new frontier. However, monotherapy often yields limited efficacy and frequent drug resistance. Future cancer treatment will involve multimodal integration to enhance overall survival rates. As emphasized by Sonkin and Thomas, Directors of the National Cancer Institute, in their authoritative review “Cancer Treatments: Past, Present, and Future” (5), identifying novel molecular targets and signaling pathways is crucial for advancing early diagnosis, prognosis assessment, and personalized therapeutic strategies.

Long noncoding RNAs (lncRNAs) are a class of non-protein-coding RNAs exceeding 200 nucleotides in length, recently identified as key regulators of gene expression and diverse biological processes. Among these, small nucleolar RNA host genes (SNHGs) constitute a unique lncRNA subclass originating from host gene sites containing snoRNAs, exhibiting distinct developmental characteristics and diverse functions. They function both as scaffolds for chromatin-modifying complexes and as competitive endogenous RNAs (ceRNAs) that sequester microRNAs, thereby playing vital roles in cancer regulatory networks (6–8). Several tumor studies have clearly demonstrated the oncogenic mechanism of “lncRNA sponge → miRNA → downstream signaling activation.” This pattern has also been echoed in research on multiple thyroid cancer-associated SNHGs (9). Furthermore, Liu et al.’s review on the prognostic, diagnostic, and therapeutic potential of gastric cancer-associated lncRNAs indicates that lncRNA research holds significant cross-cancer translational value (9).

Abnormally expressed SNHG genes play a crucial role in tumorigenesis, tumor progression, and the formation of the tumor-associated microenvironment (10, 11). In thyroid carcinoma, certain SNHG family members (e.g., SNHG7, SNHG15, SNHG12, SNHG14) have been reported as oncogenes, while others (e.g., SNHG3, SNHG5) may function as tumor suppressors—a discrepancy warranting further investigation. This review aims to systematically summarize research progress on SNHGs in thyroid cancer. We integrate the regulatory roles of different SNHGs in cellular proliferation, invasion, epithelial-mesenchymal transition (EMT), apoptosis, and drug resistance, and explore potential future research directions.

## 2 Research progress on SNHG7 and thyroid cancer

Small nucleolar RNA host gene 7 (SNHG7) is a novel oncogenic lncRNA located on chromosome 9q34.3 (12). Multiple studies have confirmed that SNHG7 is significantly overexpressed in thyroid cancer tissues and cell lines (13–17), with its upregulation closely associated with increased tumor diameter, TNM staging, and poor patient prognosis (14, 15). Functional experiments further indicate that silencing SNHG7 significantly inhibits proliferation, migration, and invasion of thyroid cancer cells while promoting apoptosis (13–15, 17), suggesting its pivotal role in thyroid cancer development.

Regarding molecular mechanisms, various studies have revealed that SNHG7 regulates multiple signaling pathways through competitive endogenous RNA (ceRNA) networks (**Table 1**). Guo et al. reported that SNHG7 can relieve the inhibition on ACSL1 by binding to miR-449a, thereby upregulating ACSL1 and promoting tumor cell growth and metastasis (13). Wang et al. discovered that SNHG7 positively regulates BDNF, further driving tumor progression (14). Chen et al. demonstrated that SNHG7 contains a miR-9-5p binding site; by binding miR-9-5p, it upregulates DPP4, thereby activating the PI3K/Akt signaling pathway. This not only promotes cell proliferation but also enhances resistance to I131 therapy (16). Furthermore, research by Chang et al. indicated that SNHG7 negatively correlates with miR-331-3p. Overexpression of SNHG7 suppresses miR-331-3p-mediated downregulation of cyclin D1 and inhibition of epithelial-mesenchymal transition (EMT), thereby accelerating tumor progression (17).

**Table 1:** SNHGs in thyroid cancer cells.

Gene	Expression in thyroid cancer	Main axis/pathway	Main phenotype	Notes
SNHG7	↑	miR-449a/ACSL1; miR-9-5p/DPP4/PI3K-Akt; miR-331-3p/Cyclin D1/EMT; BDNF	↑ Proliferation/Migration/Invasion, ↑ Radioiodine (I-131) Resistance, ↓ Apoptosis	Associated with poor prognosis; multifunctional, converges on multiple pathways.
SNHG15	↑	miR-200a-3p/YAP1 (Hippo); miR-141; Apoptosis-related proteins	↑ EMT, ↑ Migration, ↑ Proliferation, ↓ Apoptosis	Interference activates MST1/LATS1 and inhibits YAP1.
SNHG12	↑	Wnt/β-catenin; miR-16-5p	↑ Proliferation/Invasion/Metastasis, ↓ Apoptosis; ↑ G0/G1 Arrest (silencing)	Supported by GEO/TCGA + 97 paired samples; diagnostic potential.
SNHG14	↑	miR-93-5p; miR-433-3p	↑ Proliferation/Invasion/Migration, ↓ Apoptosis, ↑ EMT	Correlated with lymph node metastasis and lesion number.
SNHG5	↓	RBM47/USP21/FOXO3 (Autophagy); miR-510-5p ⊣ SNHG5	↑ Autophagy → ↓ Proliferation, ↓ Invasion/Migration	Tissue-specific tumor-suppressive signal.
SNHG3	↓	AKT/mTOR, MAPK/ERK (activated when SNHG3 is low)	Knockdown accelerates proliferation/invasion/metastasis	Tends to act as tumor suppressor.
SNHG6	↑	miR-186/CDK6	↑ Proliferation	Cell cycle–dependent pathway.
SNHG1	↑	miR-199a-5p/SP1 + positive feedback	↑ Proliferation/Invasion	SP1 promotes SNHG1 transcription.

↑, highly expressed or promoted; ↓, low expression or inhibition.

Meanwhile, bioinformatics analysis indicates that SNHG7 is closely associated with multiple cellular processes, including protein translation, RNA processing, DNA damage repair, and signaling pathway regulation (15), further supporting its multifaceted role in the initiation and progression of thyroid cancer.

In summary, SNHG7 promotes the malignant phenotype of thyroid cancer cells through multiple miRNA-related regulatory pathways and contributes to the development of radioactive iodine tolerance (13–17). Its overexpression not only indicates poor prognosis but also holds promise as a novel molecular target for the diagnosis and treatment of thyroid cancer.

## 3 Research progress on SNHG15 and thyroid cancer

Small Nucleolar RNA Host Gene 15 (SNHG15) is located on chromosome 7p13 and was first identified as a short half-life lncRNA by Tani et al. during studies on cellular stress responses (18–20). In recent years, numerous studies have demonstrated that SNHG15 is highly expressed in various tumors, including colorectal cancer (21), non-small cell lung cancer (22), breast cancer (23), and pancreatic cancer (24), and is implicated in tumorigenesis and progression.

In papillary thyroid carcinoma, Wu et al. found that SNHG15 was significantly overexpressed, with its levels closely correlated with tumor volume, advanced TNM staging, lymph node metastasis, and poor prognosis (**Table 1**) (25). Functional experiments demonstrated that silencing SNHG15 inhibited the proliferation, colony formation, and migration capabilities of thyroid cancer cells while promoting apoptosis. Concurrently, SNHG15 downregulation inhibits epithelial-mesenchymal transition (EMT), manifested by increased expression of E-cadherin and β-catenin alongside decreased expression of N-cadherin and vimentin. Further mechanistic studies revealed that SNHG15 functions as a sponge to bind miR-200a-3p, thereby upregulating its downstream gene YAP1 and promoting EMT (25). Given that YAP1 is a key downstream molecule of the Hippo signaling pathway and its overexpression is recognized as a major driver of tumorigenesis and progression (26–29), Wu et al. further demonstrated that interfering with SNHG15 activates MST1 and LATS1 within the Hippo pathway, thereby reducing YAP1 expression and inhibiting tumor progression (25).

Beyond the Hippo pathway, SNHG15 has also been reported to be closely associated with apoptosis regulation. Shuai et al. found that silencing SNHG15 inhibits cell proliferation and colony formation while promoting apoptosis, with its mechanism involving the regulation of apoptosis-related proteins such as Caspase-3, Bax, and Bcl-2 (30). Further research by Chen et al. indicated that in thyroid cancer FRO cells, downregulating SNHG15 upregulates E-cadherin and Bax while reducing Bcl-2 expression, thereby inhibiting invasion and inducing apoptosis. Conversely, SNHG15 overexpression negatively regulates miR-141 to influence the aforementioned molecular expressions, enhancing cellular proliferation and invasive capacity (31).

In summary, existing evidence indicates that SNHG15 promotes epithelial-mesenchymal transition (EMT) and tumor progression in thyroid cancer by regulating the miR-200a-3p/YAP1 axis and the Hippo signaling pathway, while simultaneously influencing the apoptosis process through miR-141 and apoptosis-related proteins (25, 30, 31). Its overexpression is closely associated with clinical malignant phenotypes, suggesting that SNHG15 not only plays a crucial role in the initiation and progression of thyroid cancer but may also serve as a potential therapeutic target and prognostic biomarker.

## 4 Research progress on SNHG12 in thyroid cancer

Small Nucleolar RNA Host Gene 12 (SNHG12) is located on chromosome 1p35.3, spans approximately 963 bp, and is widely recognized as an oncogenic lncRNA. It exhibits high expression in multiple tumors and promotes tumor progression (32, 33). In thyroid cancer, multiple studies have confirmed the abnormal upregulation of SNHG12 and its close association with clinical malignant phenotypes.

Research by Ding et al. revealed that SNHG12 is highly expressed in thyroid cancer tissues. Silencing SNHG12 inhibits cell cycle progression (G1/G0 arrest), promotes apoptosis, thereby suppressing cell proliferation, and significantly reduces cell invasion and metastasis capabilities. Animal experiments further confirmed that interfering with SNHG12 inhibits tumor cell metastasis to the lungs (34). Mechanistic studies indicate that SNHG12 regulates key molecules in the Wnt/β-catenin signaling pathway, including β-catenin, MMP-2, and Cyclin D1, thereby influencing invasion, metastasis, and proliferation in thyroid cancer cells (34). Given the well-established pivotal role of the Wnt/β-catenin pathway in the development and progression of multiple tumors, this finding suggests that SNHG12 may drive thyroid cancer progression through this pathway(**Table 1**) (35, 36).

Based on large-scale data analysis, Liu et al. utilized GEO and TCGA databases, combined with testing of 97 pairs of thyroid cancer and adjacent non-cancerous tissue samples, confirming that SNHG12 expression was significantly higher in tumor tissues than in adjacent non-cancerous tissues. Further analysis revealed that high SNHG12 expression was closely associated with lymph node metastasis and advanced TNM staging, demonstrating good sensitivity and specificity in thyroid cancer diagnosis (37).

Additionally, Feng et al. found that SNHG12 was similarly significantly upregulated in thyroid cancer tissues and cell lines (NPA87, BCPAP, and TPC1). Functional experiments demonstrated that SNHG12 promotes cell proliferation, invasion, and metastasis while inhibiting apoptosis by regulating the expression of key molecules such as PCNA, BCL-2, Bax, MMP-9, and MMP-13. Further mechanistic investigations revealed that SNHG12 acts as a competitive endogenous RNA (ceRNA) to sequester miR-16-5p, thereby reducing its expression. Related experiments validated that SNHG12’s promotion of malignant behavior in thyroid cancer cells primarily occurs through the miR-16-5p pathway (**Table 1**) (38).

In summary, SNHG12 is widely overexpressed in thyroid cancer tissues and cells, promoting tumor cell proliferation, invasion, metastasis, and anti-apoptotic processes through multiple pathways (34, 37, 38). Its abnormal upregulation is not only closely associated with adverse clinical features but also holds potential diagnostic and therapeutic applications, positioning it as a significant biomarker and molecular target for thyroid cancer.

## 5 Research progress on SNHG14 in thyroid cancer

Small Nucleolar RNA Host Gene 14 (SNHG14) is a novel long non-coding RNA (lncRNA) located on chromosome 15q11.2 that plays a crucial role in the initiation and progression of various malignant tumors. Previous studies have demonstrated that SNHG14 promotes cell proliferation and metastasis in colorectal cancer by regulating the miR-32-5p/SKIL signaling axis (39). In ovarian cancer, its overexpression is closely associated with larger tumor volume, advanced clinical stage, lymph node metastasis, and shorter overall survival. Furthermore, interfering with SNHG14 suppresses cancer cell proliferation and promotes apoptosis (40).

In thyroid cancer, Tian et al. found that SNHG14 was significantly overexpressed in thyroid cancer cell lines. Silencing SNHG14 inhibited cell proliferation, invasion, and metastasis while promoting apoptosis. Further studies confirmed that SNHG14 acts as a molecular sponge to negatively regulate miR-93-5p, thereby mediating the aforementioned biological effects (41). Ma Wenbiao et al. similarly reported significant upregulation of SNHG14 in thyroid cancer tissues, accompanied by decreased miR-433-3p levels. Functional experiments demonstrated that silencing SNHG14 inhibits cell proliferation and promotes apoptosis by regulating the cell cycle, Pro-caspase-3, and C-caspase-3. Concurrently, it suppresses the EMT process by modulating E-cadherin and N-cadherin expression, thereby reducing cell invasion and metastasis capabilities. Further validation indicated this effect depends on SNHG14’s negative regulation of miR-433-3p (42). Additionally, Tang Yue et al. found SNHG14 was significantly overexpressed in thyroid cancer tissues and cells, with its levels closely correlated with lymph node metastasis and lesion number. Silencing SNHG14 significantly inhibited the proliferation, invasion, and migration of thyroid cancer cells (TPC1) (43) (**Table 1**).

In summary, SNHG14 is widely overexpressed in thyroid cancer and promotes cell proliferation, invasion, metastasis, and suppresses apoptosis by regulating multiple miRNA-related pathways, including miR-93-5p and miR-433-3p (41–43). Its overexpression is closely associated with clinical malignant phenotypes, suggesting that SNHG14 not only plays a crucial role in the initiation and progression of thyroid cancer but may also serve as a potential molecular target and prognostic biomarker.

## 6 Research progress on SNHG5 in thyroid cancer

Small Nucleolar RNA Host Gene 5 (SNHG5) is located on human chromosome 6q15 and plays a significant role in the initiation and progression of various malignant tumors (44). Previous studies have reported that SNHG5 is highly expressed in gliomas, where it promotes glucose uptake and enhances cell migration and invasion capabilities (45). Conversely, in gastric cancer, research by Zhao et al. suggests SNHG5 may exert tumor-suppressing effects, demonstrating tissue-specific dual functionality (46).

Regarding thyroid cancer, Qin et al. observed low expression of both SNHG5 and the RNA-binding protein RBM47 in thyroid cancer tissues and cell lines. Functional experiments demonstrated that high RBM47 expression inhibits thyroid cancer cell proliferation by regulating the LC3 II/LC3 I ratio in the autophagy flux. Further studies revealed direct interaction between RBM47 and SNHG5, with SNHG5 recruiting the deubiquitinating enzyme USP21. Following interaction with USP21, FOXO3 activates autophagy, thereby inhibiting cancer cell proliferation (47). This finding suggests SNHG5 may exert tumor-suppressing effects in thyroid cancer through the RBM47/USP21/FOXO3 axis.

Additionally, studies by Gan Shaoping et al. revealed that miR-510-5p was significantly upregulated in thyroid cancer nude mouse tissues and 8505C cells, while SNHG5 expression was markedly downregulated. Functional experiments demonstrated that inhibiting miR-510-5p suppressed cell proliferation, invasion, and migration. Mechanistic studies further confirmed that miR-510-5p directly binds to and negatively regulates SNHG5, thereby affecting its functional expression in thyroid cancer cells (48) (**Table 1**).

In summary, SNHG5 is universally underexpressed in thyroid cancer and exerts tumor-suppressive effects through the RBM47/USP21/FOXO3 pathway and miR-510-5p-related mechanisms. Unlike its pro-tumorigenic function in gliomas and other tumors (45), SNHG5 may act as a tumor suppressor in thyroid cancer. This tissue-specific discrepancy suggests the complexity of its mechanisms in tumorigenesis and progression.

## 7 Research progress on SNHG3 in thyroid cancer

Small Nucleolar RNA Host Gene 3 (SNHG3) is a novel long non-coding RNA (lncRNA) located on human chromosome 1p36.1. Initial studies revealed that abnormal expression of SNHG3 is closely associated with the onset of Alzheimer’s disease (49). Subsequently, researchers identified abnormal SNHG3 expression in multiple malignant tumors and demonstrated its oncogenic role in certain cancers. For instance, elevated SNHG3 promotes osteosarcoma cell invasion and metastasis by regulating the miR-151a-3p/RAB22A signaling axis (50). In bladder cancer, Dai et al. demonstrated that high SNHG3 expression correlates with shorter overall survival, larger tumor diameter, and increased risk of distant metastasis (51).

In contrast to its oncogenic role in the aforementioned tumors, SNHG3 exhibits downregulation in thyroid cancer. Duan et al. found significantly reduced SNHG3 expression in thyroid cancer tissues and cell lines (BCPAP, TPC1, and KTC1), with expression levels correlating closely with patient TNM staging. Functional experiments further confirmed that SNHG3 knockdown significantly enhanced the proliferation, invasion, and metastatic capacity of thyroid cancer cells (52) (**Table 1**).

Extensive research indicates that the AKT/mTOR and MAPK/ERK signaling pathways play crucial roles in the initiation and progression of malignant tumors, with their activation significantly enhancing cancer cell proliferation, invasion, and metastasis (53–55). Against this backdrop, Duan et al. identified through gene enrichment analysis that SNHG3 downregulation is closely associated with the activation of mTOR and MAPK pathways. Further molecular mechanism studies revealed that SNHG3 knockdown upregulates key molecules in the AKT/mTOR and MAPK/ERK signaling pathways (p-AKT, p-mTOR, and p-ERK), thereby accelerating thyroid cancer progression (52).

In summary, SNHG3 exhibits tissue-specific dual roles across different tumors. In osteosarcoma and bladder cancer, SNHG3 acts as an oncogenic factor (50, 51); whereas in thyroid cancer, SNHG3 may function as a tumor suppressor gene by negatively regulating the AKT/mTOR and MAPK/ERK signaling pathways (52). This discrepancy suggests that the mechanisms underlying SNHG3’s role in tumorigenesis and progression are complex and tissue-specific.

## 8 Research progress on SNHG6 in thyroid cancer

Small Nucleolar RNA Host Gene 6 (SNHG6), also known as U87HG, is a novel long non-coding RNA (lncRNA) localized to chromosome 8q13.1. Studies have demonstrated that SNHG6 is highly expressed in various malignancies, including lung cancer (56), colorectal cancer (57), and hepatocellular carcinoma (58), where it exerts pro-cancerous effects. Xu et al. (59) analyzed the UALCAN database and found that SNHG6 was significantly overexpressed in thyroid cancer tissues, with its levels closely correlated to patients’ clinical staging. Compared to normal thyroid cells (Nthy-ori-3-1), SNHG6 was also highly expressed in thyroid cancer cell lines K1 and TPC1. Functional experiments demonstrated that interfering with SNHG6 significantly inhibited thyroid cancer cell proliferation, while SNHG6 overexpression enhanced cellular proliferation capacity.

Regarding mechanism studies, the Starbase database predicted potential binding sites for miR-186 on SNHG6. Firefly luciferase reporter assays further validated that miR-186 directly binds to SNHG6. Notably, CDK6 is a cell cycle-dependent kinase playing a crucial role in G1 phase progression and G1/S transition. It is highly expressed in various malignant tumors and promotes cell proliferation (60–62). Xu et al. (59) further demonstrated that SNHG6 negatively regulates miR-186 by acting as a molecular sponge, thereby releasing its suppression of the downstream target gene CDK6. This leads to elevated CDK6 expression and ultimately promotes the proliferation of thyroid cancer cells (**Table 1**).

## 9 Research progress on SNHG1 and thyroid cancer

Small Nucleolar RNA Host Gene 1 (SNHG1) is a newly discovered long non-coding RNA (lncRNA) located on human chromosome 11q12.3. SNHG1 is highly expressed in various malignant tumors and plays a crucial role in tumorigenesis and progression (63–65). In thyroid cancer, Ding et al. (74) found that SNHG1 was highly expressed in thyroid cancer tissues compared to normal tissues, with its levels significantly correlated with tumor size. Compared to normal thyroid cells (Nthy-ori-3-1), SNHG1 was also highly expressed in thyroid cancer cell lines IHH4, K1, and TPC1. Functional experiments demonstrated that silencing SNHG1 significantly inhibited the proliferation and invasion of thyroid cancer cells.

Mechanistic studies revealed that SNHG1 directly targets and negatively regulates miR-199a-5p, thereby releasing its inhibitory effect on the downstream target gene SP1 and leading to increased SP1 expression levels. Notably, SP1 binds to the SNHG1 promoter region, enhancing SNHG1 transcriptional activity and forming a positive feedback regulatory loop involving SP1/SNHG1/miR-199a-5p, which further amplifies pro-cancer signaling (66). This finding suggests that SNHG1 promotes thyroid cancer cell proliferation and invasion by establishing a stable positive feedback network, potentially positioning it as a key molecular target for future thyroid cancer therapies (**Table 1**).

## 10 Discussion

Through the aforementioned studies on SNHG, we can observe that these molecules share certain common mechanisms in thyroid cancer. Taking SNHG7, SNHG15, SNHG12, and SNHG14 as examples, although they act on different miRNAs, they ultimately activate signaling pathways such as PI3K/Akt, Wnt/β-catenin, or Hippo–YAP1 by competitively binding to miRNAs, thereby promoting epithelial-mesenchymal transition (EMT) and cellular invasion and metastasis. This phenomenon suggests that multiple SNHGs may converge onto similar signaling networks at different molecular entry points. Therefore, in therapeutic exploration, inhibitors targeting EMT or the aforementioned key signaling pathways, or nucleic acid-based drugs directly blocking SNHG–miRNA interactions, may simultaneously intervene in the oncogenic effects of multiple SNHGs, demonstrating synergistic potential.

Concurrently, not all SNHGs exhibit consistent oncogenic properties. SNHG3 and SNHG5 show relatively low expression in thyroid cancer and possess certain inhibitory effects, closely linked to their roles in autophagy regulation (the SNHG5–RBM47/USP21–FOXO3 axis) and modulation of AKT/mTOR/MAPK signaling pathways. Compared to oncogenic SNHGs, the low expression of these molecules may reflect active suppression of tumor suppressor signaling by tumor cells. This functional differentiation suggests that SNHG family members are not monomodal and may assume opposing roles in different tissues or molecular environments. Investigating the upstream regulatory mechanisms underlying these differences—such as transcriptional control, epigenetic modifications, and RNA-binding protein involvement—could elucidate the bidirectional effects of distinct SNHGs.

Furthermore, to better understand SNHG functions, they must be contextualized within broader cancer biology frameworks. For instance, existing studies indicate that non-coding RNAs are closely associated with inflammatory signaling and stress responses (67), suggesting SNHGs may indirectly promote thyroid cancer progression through the inflammatory microenvironment. Similarly, infection-driven cancer migration mechanisms (68) offer insights into how SNHGs regulate epithelial-mesenchymal transition (EMT) and metastasis.

## 11 Conclusion

In summary, the SNHG family exhibits both convergent mechanisms and functional differences. Shared mechanisms provide a basis for identifying common therapeutic targets, while functional variations suggest the potential value of SNHG in personalized treatment and prognostic assessment. Future research should not only delve deeper into molecular mechanisms but also design experiments that more closely mimic clinical scenarios. Enhanced integration with fields such as inflammation, ion channels, and traditional medicine is essential. Only through such expansive studies can we fully unravel the role of SNHG in thyroid cancer development and treatment, thereby providing novel targets and strategies for precision medicine.
